# A QUALITATIVE ANALYSIS OF UNDERGRADUATE STUDENT AND OLDER ADULT REFLECTIONS ON AN ORAL HISTORY PROJECT

**DOI:** 10.1093/geroni/igad104.3147

**Published:** 2023-12-21

**Authors:** Gabriele Moriello, Katie Ehlman, Mary Ligon

**Affiliations:** Utica University, Utica, New York, United States; University of Southern Indiana, Evansville, Indiana, United States; York College of Pennsylvania, Grantley, Pennsylvania, United States

## Abstract

Interaction between generations is diminishing as extended family members live further apart and as neighborhoods, communities, and institutions become increasingly age-segregated. Fostering intergenerational contact can benefit both parties involved. The aim of this qualitative study was to develop a deeper understanding of the meaning of participating in an intergenerational oral history project using a phenomenological approach. Undergraduate students (n=21) interviewed a family member or friend in three, 45-60-minute sessions. After the last interview, each older adult/student pair met to share what the project meant to them. Audio recordings of these final reflections were analyzed using a thematic analysis process. Five themes emerged: Reflection, Learning, Meaningful Connections, Evoked Emotions, and Advice. To the surprise of the researchers, the five themes were common to both students and older adults. The subthemes, however, differed between groups within three of the themes (reflection, learning, and advice). Consistent with previous research, findings support that participating in an oral history project is beneficial for both parties. This study adds to the literature by revealing that both older adults and students reflected on their lives. Older adults tended to accept their lives (or not) after reflecting. Both groups learned as a result of this project and the project evoked emotions. Theoretical study implications include that engaging in reminiscence supports psychological development in late-life, according to Erikson’s model. Further, congruent with Mezirow’s theory of transformative learning, the experience challenged participants to consider how telling and/or hearing a life story fits or did not fit with prior thoughts.

